# Mechanical loading-induced change of bone homeostasis is mediated by PGE2-driven hypothalamic interoception

**DOI:** 10.21203/rs.3.rs-3325498/v1

**Published:** 2023-09-15

**Authors:** Feng Gao, Qimiao Hu, Cheng Qi, Mei Wan, James Ficke, Junying Zheng, Xu Cao

**Affiliations:** 1 Department of Orthopedic Surgery and Department of Biomedical Engineering, Johns Hopkins University School of Medicine, Baltimore, Maryland 21205, USA.

## Abstract

Bone is a mechanosensitive tissue and undergoes constant remodeling to adapt to the mechanical loading environment. However, it is unclear whether the signals of bone cells in response to mechanical stress are processed and interpreted in the brain. In this study, we found that the hypothalamus of the brain regulates bone remodeling and structure by perceiving bone PGE2 concentration in response to mechanical loading. Bone PGE2 levels are in proportion to their weight bearing. When weight bearing changes in the tail-suspension mice, the PGE2 concentrations in bones change in line with their weight bearing changes. Deletion of *Cox2* or *Pge2* in the osteoblast lineage cells or knockout *Ep4* in sensory nerve blunts bone formation in response to mechanical loading. And sensory denervation also significantly reduces mechanical load-induced bone formation. Moreover, mechanical loading induces CREB phosphorylation in the hypothalamic ARC region to inhibit sympathetic TH expression in the PVN for osteogenesis. Finally, we show that elevated PGE2 is associated with ankle osteoarthritis (AOA) and pain. Together, our data demonstrate that in response to mechanical loading, skeletal interoception occurs in the form of hypothalamic processing of PGE2-driven peripheral signaling to maintain physiologic bone homeostasis, while chronically elevated PGE2 can be sensed as pain during AOA and implication of potential treatment.

## Introduction

The primary functions of the skeleton of terrestrial animals are to provide mechanical support for locomotion and physical activity, to protect fragile organs and to serve as a metabolic mineral reserve(1). The mechanical properties of bone are dependent on its microarchitecture and its integrity is critical to prevent fracture(2). The endocrine regulation of bone metabolism, including the balance between osteoclast-mediated bone catabolism and osteoblast-induced bone anabolism, has been extensively studied in recent decades(3–6). Aberrant mechanical loading induces uncoupled bone remodeling to generate porosity structure of spine endplate for low back pain and porous subchondral bone for osteoarthritis pain(7–9). Currently, there is no disease-modifying treatment for the joint pain orders, leaving surgical treatments such as joint replacement or spinal fusion as only approaches for end stage of these diseases(10–12). Bone is a mechanosensitive tissue that undergoes constant remodeling to adapt to the physical environment, including loading-induced microfractures that repeatedly occur in response to gravity and subsequent weight bearing(13). Mechanobiology, in general, has been extensively studied at the cellular and molecular levels, including the role of cell membrane adhesion molecules, receptors and different ion channels in the process(14–16). However, it is still unclear whether the signals derived from bone cells in response to mechanical stress are processed and interpreted in the brain to regulate bone remodeling to maintain its mechanical structure and metabolism during normophysiology and how this might go awry during disease.

Mechanical loads increase bone formation by stimulating the activity of bone-forming osteoblasts(17). In contrast, loss of mechanical loads induces negatively balanced bone remodeling, resulting in more bone resorption than bone formation, like astronauts in space(18). Bone cells are tightly coupled to their extracellular environment and coordinate the bone formation process by converting external mechanical load into biochemical responses, which is known as mechanotransduction(19). The external mechanical load generates deformation in the bone tissue, which results in changes in whole tissue strain, shear stress, hydrostatic pressure, and streaming potentials generated by bone fluid flow(20). Many mechanisms that mediate mechanotransduction have been studied extensively in various experimental models(21–24). PGE2 is considered a potent anabolic regulator of bone growth. The PGE2 level increases in loaded bone tissue, and direct administration results in bone formation(25). PGE2 administration also increases the sensitivity of bone to mechanical loading(26). Biochemical blockage of prostaglandins leads to bone’s inability to sense and respond to mechanical stimulation(27). Moreover, osteoblasts subjected to fluid shear increase the expression of cyclooxygenase-2 (COX-2), the enzyme responsible for PGE2 synthesis(28). Taken together, PGE2 serves as a mechanotransduction signal that translates the external mechanical load into biochemical responses.

Interoception represents and monitors the organism’s internal state to regulate the complex interactions between the brain and peripheral organs(29, 30). The signals collected from sensory nerve endings of peripheral tissues, such as skin, joint, respiratory, and gastrointestinal tissues, are processed in CNS to initiate physiological responses. The hypothalamus controls whole-body homeostasis by integrating peripheral information and coordinating peripheral organs through descending neural or neuroendocrine pathways(31). We have recently established that skeletal interoception regulates bone homeostasis. The sensory nerve is activated by bone PGE2 to induce phosphorylation of CREB in the hypothalamus as ascending interoceptive signal, which tunes down sympathetic activity for osteoblastic bone formation as the descending interoceptive signal(32, 33). Importantly, the skeletal interoceptive signal also downregulates hypothalamic Neuropeptide Y (NPY) expression to induce adipose tissue lipolysis for osteoblastic bone formation(34). Moreover, skeletal interoception promotes biomaterial-mediated new bone formation through divalent metal cations stimulation of macrophage secretion of PGE2 pain(35). These results suggest that PGE2 skeletal interoception regulates bone quality in response to mechanical loading.

To date, the PGE2 skeletal interoception in regulating bone remodeling has been mostly studied in the pathophysiological conditions(36). It is still unclear whether the PGE2 skeletal interoception in response to mechanical stress is processed during normal physiology and how the brain interprets the signaling. In this study, we found from the naïve C57BL/6 mice of 12-weeks-old, the density of different bones in the body is proportionally correlated to PGE2 concentrations in the bone marrow. Notably, we found that the talus, which bears more body weight than any other weight-bearing bones during movement or physical activities, has the highest PGE2 levels. Mechanistically, we found that mechanical loading increased osteogenesis by increasing CREB phosphorylation in the arcuate nucleus of the hypothalamus, which resulted in the suppression of sympathetic tone. On a molecular level, such PGE2-driven interoception resulted in an elevation of inducible cAMP early repressor (ICER), the endogenous repressor of cAMP-responsive element (CRE)-mediated gene transcription, to suppress TH gene expression and thus sympathetic tone. Finally, we found that elevated PGE2 levels are positively associated with ankle osteoarthritis-related pain. COX2 expression in the talus was extremely high, which explains painful ankle osteoarthritis and PGE2 in control of bone quality and skeletal pain.

## Results

### PGE2 concentrations in various bones are positively correlated with their degree of weight bearing

**Figure 1.**
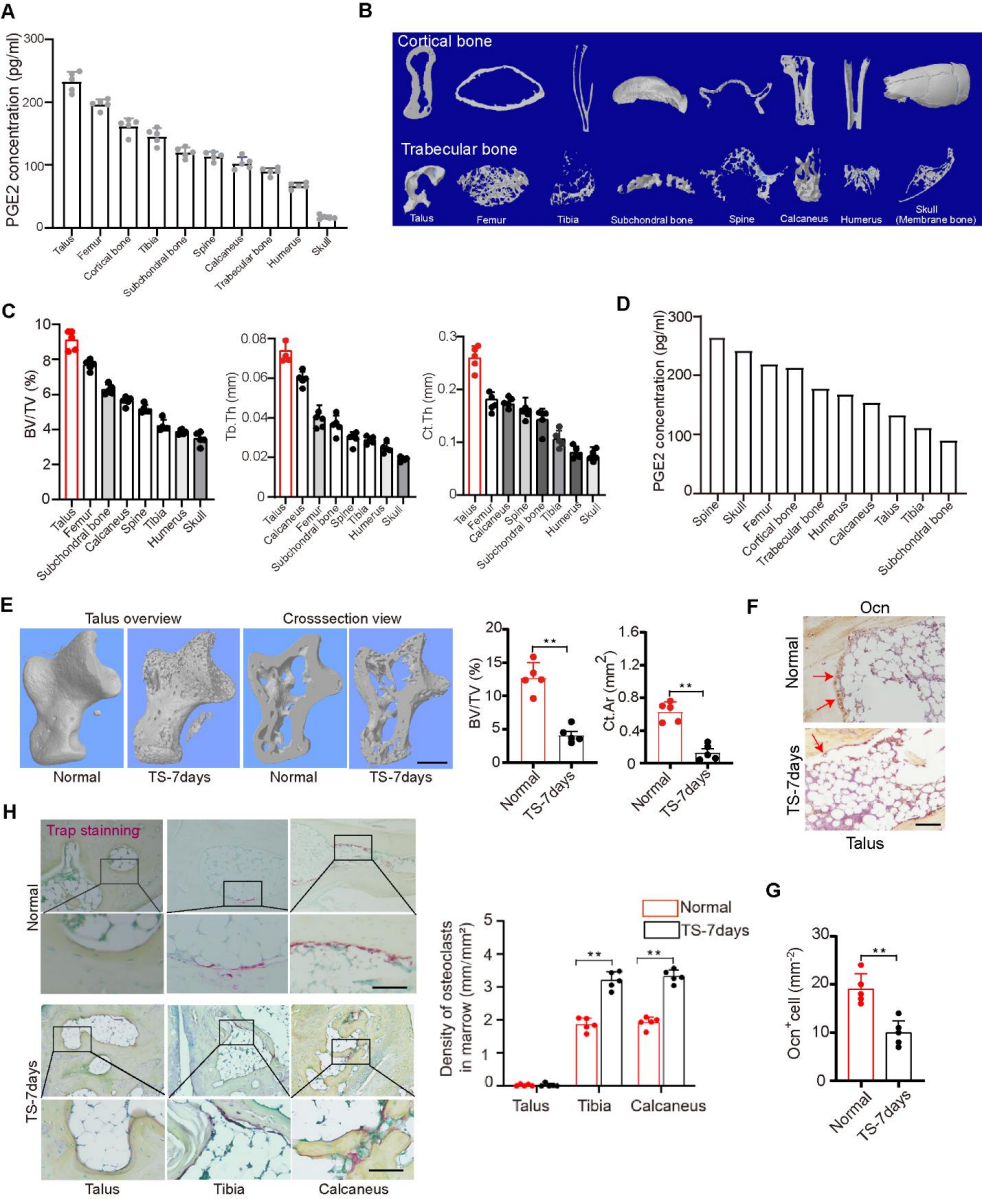
PGE2 concentration in bone is positively correlated with mechanical load. (A)Enzyme-linked immunosorbent assay (ELISA) analysis of PGE2 level in bone at 10 different points of 12-week-old C57BL/6 mice. (B) Representative μCT images of 8 different point bones from 12-week-old C57BL/6 mice. (C) Quantitative analysis of trabecular bone fraction (BV/TV), trabecular bone thickness (Tb. Th) and cortical bone thickness (Ct. Th). (D)ELISA analysis of PGE2 level in bone at 10 different points of 13-week-old C57BL/6 mice with tail suspension (TS) for 7 days. (E) Representative μCT images and quantitative analysis of trabecular bone fraction (BV/TV), cortical bone area (Ct. Ar) of talus from normal 13-week-old C57BL/6 mice or with tail suspension (TS) for 7 days. Scale bars, 50 μm. (F and G) Representative images of immunostaining of osteocalcin (Ocn) positive cells (F) and analysis of Ocn+ cells in the subchondral bone of talus from normal 13-week-old C57BL/6 mice or with tail suspension (TS) for 7 days (G). Scale bars, 50 μm. (H) Representative images of immunostaining and quantitative analysis of the density of TRAP+ cells in the subchondral bone of talus, tibia, and calcaneus from normal 13-week-old C57BL/6 mice or with tail suspension (TS) for 7 days. Scale bars, 50 μm. N ⩾ 5 per group. *P < 0.05, **P<0.01, and N.S. indicates not significant. Statistical significance was determined by Student’s t-test.

Mechanical loading stimulates an increase in PGE2 levels in the skeletal system. To investigate the role of this increase in mechanical loading, we measured PGE2 levels in the bone at ten different locations of C57BL/6 mice at 12 weeks of age. Interestingly, PGE2 levels in the talus were the highest and lowest in the skull (Figure 1A). Analysis by *in vitro* microcomputerized tomography (μCT) of bones at different locations within the body harvested at 12 weeks of age revealed that the talus had the highest bone volume fraction (BV/TV), as well as the trabecular thickness (Tb.Th) and cortical thickness (Ct.Th), which are indices of trabecular and cortical structure, respectively, while the skull had the lowest of these three parameters. (Figure 1B, C). Together, these findings indicate that concentration of PGE2 in a given site of bone is directly proportional to its degree of mechanical loading and its microarchitecture.

To determine whether the change of mechanical loading in the skeleton affects PGE2 secretion to regulate bone homeostasis, tail suspension (TS) was investigated. TS is a ground-based model that creates hypokinesia and hypodynamia at the hind limbs and mimics cephalic fluid-shift aspects of space flight. Extensive studies in rats showed that it induces both cortical and cancellous bone loss in the hind limbs(37). We thus applied TS to C57B/L6 mice and measured bone PGE2 levels. We found that PGE2 levels significantly were decreased in the talus compared with the grounded control group, while it was increased in the skull and spine (Figure 1D). Bone mass and cortical bone of the talus decreased significantly in TS mice while body weight remained unchanged over time relative to their grounded littermate controls (Figure 1E). Again, the number of osteoblasts in the talus decreased significantly in the TS mice compared to the controls (Figure 1F, G), whereas the number of TRAP+ osteoclasts in the talus were not detected either in the ground control nor the TS mice (Figure 1H). As PGE2 is predominantly secreted by osteoblasts, the decrease of osteoblasts is consistent with the decrease of PGE2 levels in the talus after 7 days of TS.

### PGE2 concentrations in the bone increase in response to mechanical loading to regulate bone formation

**Figure 2.**
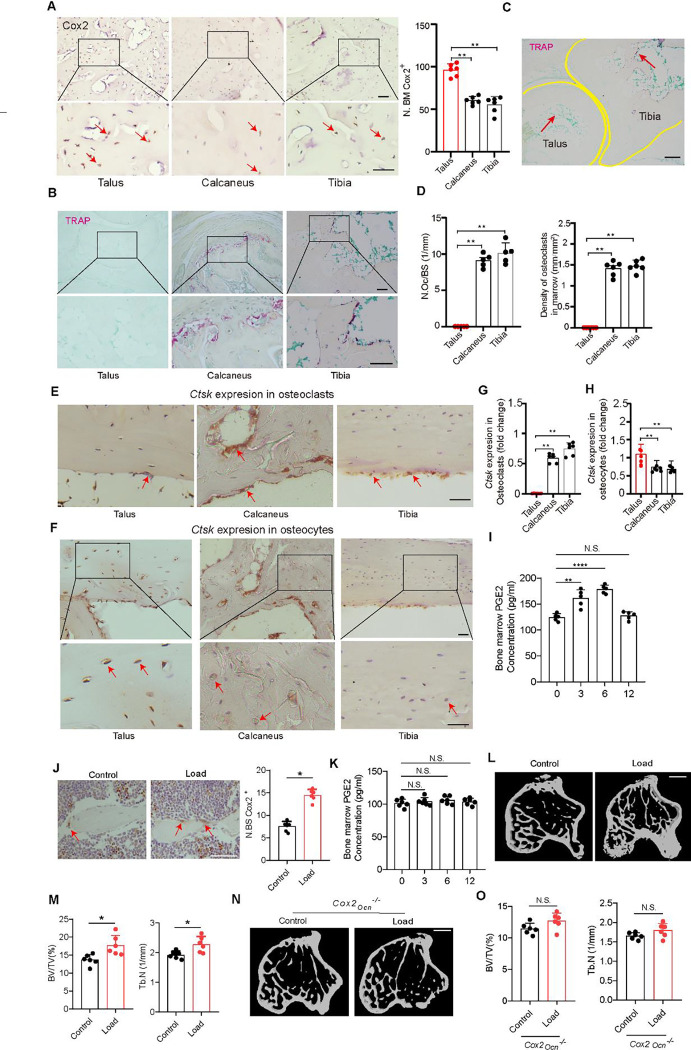
PGE2 mediates mechanical load-induced osteoblastic bone formation. (A) Representative images of immunostaining and quantitative analysis of the Cox2+ cells (brown) in the subchondral bone of talus, calcaneus, and tibia from 12-week-old C57BL/6 mice. Scale bars, 50 μm. (B-D) Representative images of immunostaining TRAP+ cells per bone surface (N.Oc/BS) on the trabecular bone surface (B) and TRAP+ cells in the bone marrow (C) and quantitative analysis of TRAP+ cells (D) in talus, calcaneus, and tibia from 12-week-old C57BL/6 mice. Scale bars, 50 μm. (E, G) Representative images of immunostaining (E) and quantitative analysis (G) of Ctsk expression in osteoclasts at steady state of the subchondral bone of talus, calcaneus, and tibia from 12-week-old C57BL/6 mice. Scale bars, 50 μm. (F, H) Representative images of immunostaining (F) and quantitative analysis (H) of Ctsk expression in osteocytes at steady state of the subchondral bone of talus, calcaneus, and tibia from 12-week-old C57BL/6 mice. Scale bars, 50 μm. (I) ELISA analysis of PGE2 level in tibiae bone marrow at different time points after axial compression loading on the tibiae 100 cycles at 2 Hz of WT mice. (J-O) Mice underwent one month of axial compression loading of the tibiae. Non-loaded tibiae were used as controls. (J) Immunohistochemical staining and quantification of Cox2^+^ cells (brown) on the trabecular tibial surface in WT mice. Scale bar, 50 μm. (K) ELISA analysis of PGE2 level in tibiae bone marrow at different time points after axial compression loading on the tibiae 100 cycles at 2 Hz of Cox2_*Ocn*_^−/−^ mice. (L, M) Representative μCT images (L) and quantitative analysis (M) of trabecular bone fraction (BV/TV) and trabecular number (Tb.N) of tibial bone of WT mice loaded for one month or non-loaded tibiae. Scale bar, 500 μm. (N, O) Representative μCT images (N) and quantitative analysis (O) of trabecular bone fraction (BV/TV) and trabecular number (Tb.N) of tibial bone of Cox2_Ocn_^−/−^ mice loaded for one month or non-loaded tibiae. Scale bar, 500 μm. N ⩾ 5 per group. *P < 0.05, **P<0.01, and N.S. indicates not significant. Statistical significance was determined by Student’s t-test.

We next sought to examine whether Cox2, which is the key rate-limiting biosynthetic enzyme for PGE2, mediates bone formation. By analysis of Cox2 expression levels, we found significantly higher expression in the talus than in the tibia and calcaneus (Figure 2A). Of note, we found that there was practically no tartrate-resistant acid phosphatase (TRAP) staining in the talus while there was relatively strong staining in the tibia and calcaneus of adult mice (Figure 2B), which correlated with a strong difference in the number of osteoclasts per bone surface (N.Oc/BS) and the density of osteoclasts in the bone marrow of these bones (Figure 2C and D). We also found that cathepsin K (Ctsk), which is highly expressed and secreted by osteoclasts and is required for their bone-resorbing activity, is also expressed in osteocytes (Figure 2F). Notably, we observed a considerable number of Ctsk^+^ osteoclasts at steady state in the calcaneus and tibia, but not in the talus (Figure 2E, G), while immunohistochemistry revealed Ctsk^+^ osteocytes at steady state in the talus, calcaneus and tibia (Figure 2F, H). These results likely indicate that Ctsk in osteocytes regulates talus bone surface remodeling. These findings provide evidence that under physiological conditions, PGE2 in the talus mediates bone remodeling independent of osteoclasts.

PGE2 is known to stimulate osteoblastic bone formation. We measured PGE2 levels in the tibia bone marrow (Figure 2I) following axial tibial compression. We found that PGE2 levels increased at 3 and 6 h after loading and then dropped to normal levels after 12 h (Figure 2I). By immunohistochemical analysis we also found that expression of Cox2 was higher on the trabecular surface of the tibial bone after loading compared to the unload control (Figure 2J). In addition, by μCT analysis we found that there was a significantly greater degree of bone formation after loading for 1 month (Figure 2L, M).

To further examine whether PGE2 is secreted primarily by osteoblastic cells in response to mechanical loading, we generated conditional knockout *Cox2* mice in osteoblastic cells (*Cox2*_*Ocn*_^−/−^) by crossing *Cox2* floxed (*Cox2*^*wt*^) mice with osteocalcin (*Ocn*)-Cre mice to eliminate PGE2 secretion by osteoblastic cells as Ocn is a selective marker of osteoblasts. Bone PGE2 concentrations were not changed after loading within 12 h in the *Cox2*_*Ocn*_^−/−^ mice, suggesting that PGE2 was largely secreted by osteoblasts (Figure 2K). Moreover, we did not see an increase in bone formation in *Cox2*_*Ocn*_^−/−^ mice after mechanical loading (Figure 2N, O) suggesting that mechanical loading via upregulation of PGE2 levels promotes bone formation.

### Sensory nerve denervation reduces talus bone formation

**Figure 3.**
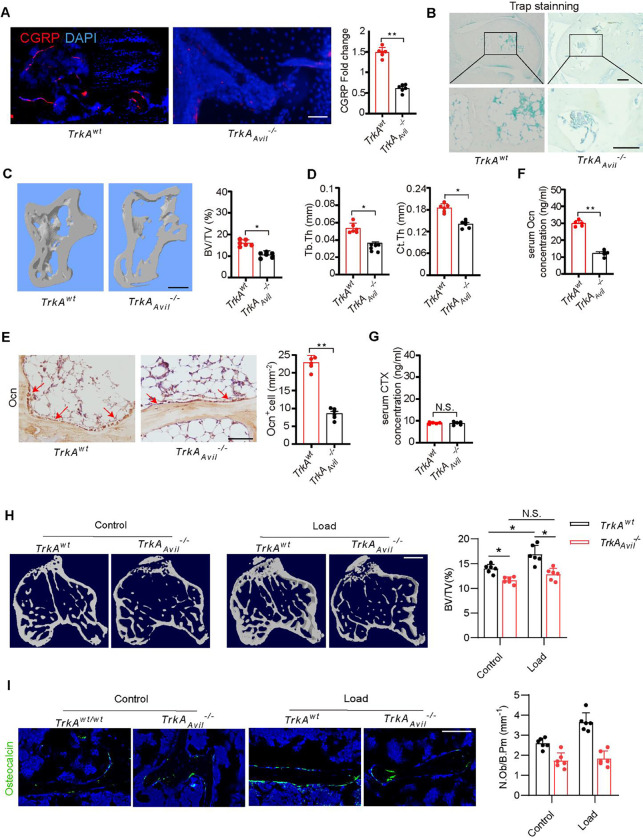
Sensory denervation reduces mechanical load-induced bone formation. (A) Representative images of immunofluorescence staining and quantitative analysis of the CGRP+ sensory nerves (red) in the subchondral bone of talus from 12-week-old *TrkA*^*wt*^ and *TrkA*_*Avil*_^−/−^ mice. Scale bar, 100 μm. (B) Representative images of immunostaining of TRAP+ cells in the subchondral bone of talus from 12-week-old *TrkA*^*wt*^ and *TrkA*_*Avil*_^−/−^ mice. Scale bar, 100 μm. (C, D) Representative μCT images and quantitative analysis of trabecular bone fraction (BV/TV) (C), trabecular bone thickness (Tb. Th) and cortical bone thickness (Ct. Th) (D) of talus from 12-week-old *TrkA*^*w*t^ and *TrkA*_*Avil*_^−/−^ mice. Scale bar, 100 μm. (E) Representative images of immunostaining of Ocn and analysis of Ocn+ cells in the subchondral bone of talus from 12-week-old *TrkA*^*wt*^ and *TrkA*_*Avil*_^−/−^ mice. Scale bar, 100 μm. (F, G) ELISA analysis of Ocn (F) and carboxy-terminal collagen crosslinks (CTX) level of the serum (G) from 12-week-old *TrkA*^*w*t^ and *TrkA*_*Avil*_^−/−^ mice. (H, I) *TrkA*^*wt*^ and *TrkA*_*Avil*_^−/−^ mice underwent one month of axial compression loading of tibiae. Non-loaded tibiae were used as controls. (H) Representative μCT images and quantitative analysis of trabecular bone fraction (BV/TV) of tibial bone. Scale bar, 500 μm. (I) Representative images of immunofluorescence staining and quantitative analysis of Ocn+ cells (green) on trabecular bone surface of tibiae. Scale bar, 50 μm. N ⩾ 5 per group. *P < 0.05, **P<0.01, and N.S. indicates not significant. Statistical significance was determined by Student’s t-test.

To investigate the role of sensory nerves in talus homeostasis, we crossed nerve growth factor receptor *TrkA* floxed (*TrkA*^*wt*^) mice with sensory neuron-specific cre mice (*Advilin*-Cre) to generate mice with sensory denervation (*TrkA*_*Avil*_^−/−^). Immunostaining of talus sections showed that most calcitonin gene-related peptide (CGRP)^+^ sensory nerve fibers were eliminated in the *TrkA*_*Avil*_^−/−^ mice (Figure 3A). TRAP staining showed no osteoclast formation in either the talus of the *TrkA*^*wt*^ or *TrkA*_*Avil*_^−/−^ mice (Figure 3B). By μCT analysis we found that there was significantly less bone in talus in 12-week-old *TrkA*_*Avil*_^−/−^ mice relative to their wild-type (WT) littermates (Figure 3C). In addition, Tb.Th and Ct.Th were lower in *TrkA*_*Avil*_^−/−^ mice compared to *TrkA*^*wt*^ mice indicating an essential role of sensory nerve function for talus homeostasis in adults (Figure 3D). Notably, the number of Ocn^+^ osteoblasts and the serum levels of OC were significantly lower in *TrkA*_*Avil*_^−/−^ mice relative to their wild-type littermates (Figure 3E and F), while the level of the osteoclast bone resorption marker, carboxy-terminal collagen crosslinks (CTX), was not different in *TrkA*_*Avil*_^−/−^ mice compared to *TrkA*^*wt*^ control (Figure 3G).

### Sensory nerve denervation reduces mechanical loading-induced bone formation

We next tested whether sensory nerve denervation could reduce mechanical loading-induced bone formation. *TrkA*^*wt*^ and *TrkA*_*Avil*_^−/−^ mice underwent one month of axial compression loading of tibiae. In the *TrkA*^*wt*^ mice, mechanical load significantly increased the bone formation in the tibiae compared to the non-loaded controls (Figure 3H). The load induced bone formation was abolished by sensory nerve denervation in the *TrkA*_*Avil*_^−/−^ mice (Figure 3H). In addition, immunostaining of trabecular bone sections showed that Ocn^+^ osteoblastic cells were significantly greater in wild-type mice after mechanical loading compared to the unload control, and no such increase occurred in *TrkA*_*Avil*_^−/−^ mice (Figures 3I). Taken together, these results indicate that peripheral sensory nerves regulate load-induced osteogenesis.

### Mechanical loading induces osteogenesis via central CREB signaling

**Figure 4.**
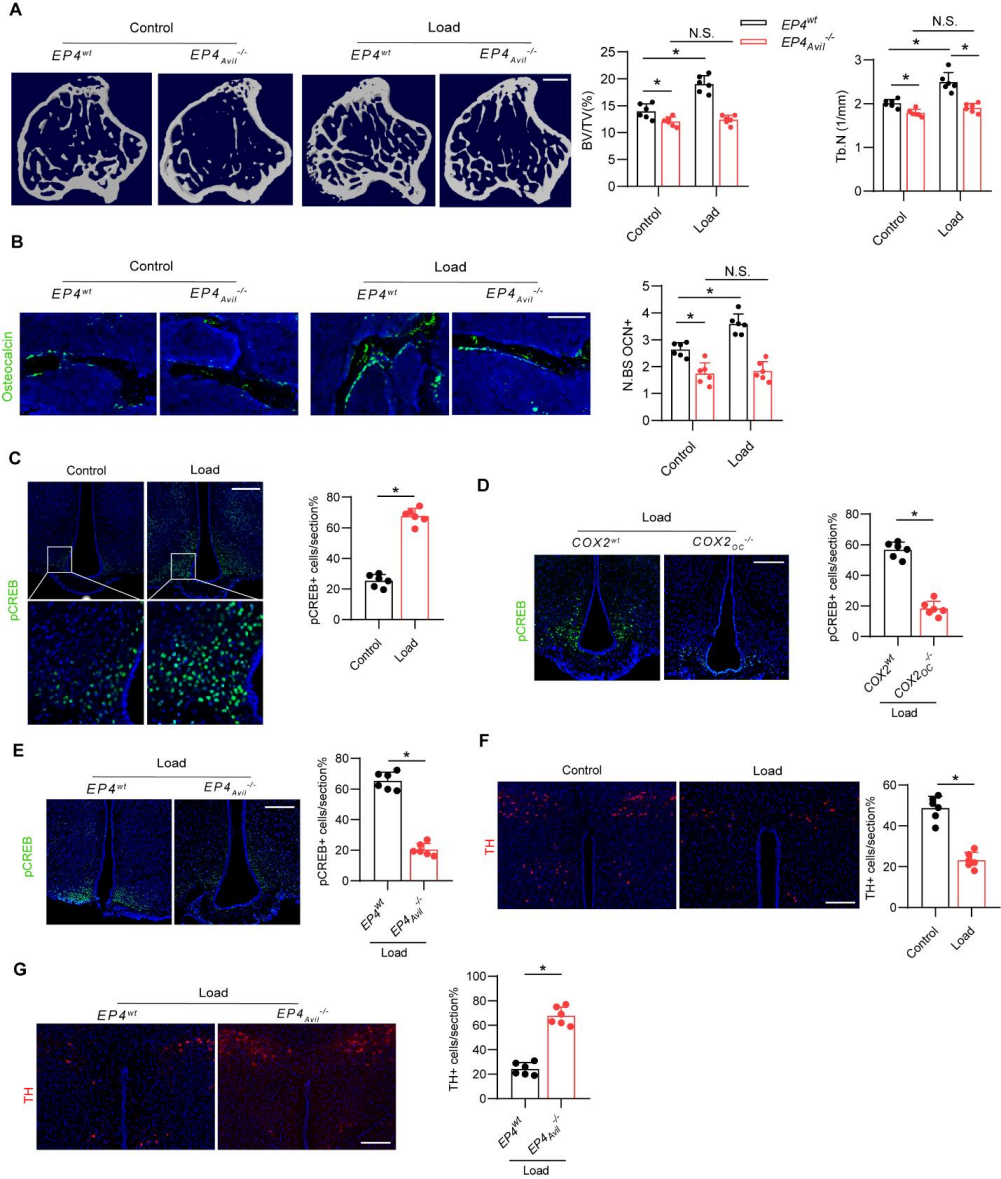
Mechanical load induces osteogenesis through PGE2/EP4 interoceptive signaling. (A, B) *EP4*^*wt*^ and *EP4*_*Avil*_^−/−^ mice underwent one month of axial compression loading of tibiae. Non-loaded tibiae were used as controls. (A) Representative μCT images and quantitative analysis of trabecular bone fraction (BV/TV) and trabecular number (Tb.N) of tibial bone. Scale bar, 500 μm. (B) Representative images of immunofluorescence staining of Ocn and quantitative analysis of Ocn+ cells (green) on trabecular bone surface of tibiae. Scale bar, 50 μm. (C) Representative images of immunofluorescence staining and quantitative analysis of the pCREB+ cells in the ARC of the hypothalamus of WT mice underwent three consecutive days of axial compression loading of tibiae or control shame load. Scale bar, 50 μm. (D) Representative images of immunofluorescence staining and quantitative analysis of the pCREB+ cells in the ARC of the hypothalamus of *COX2*^*wt*^ and *COX2*_*Ocn*_^−/−^ mice underwent three consecutive days of axial compression loading of tibiae. Scale bar, 50 μm. (E) Representative images of immunofluorescence staining and quantitative analysis of the pCREB+ cells in the ARC of the hypothalamus of *EP4*^*wt*^ and *EP4*_*Avil*_^−/−^ mice underwent three consecutive days of axial compression loading of tibiae. Scale bar, 50 μm. (F) Representative images of immunofluorescence staining and quantitative analysis of the TH+ cells in the PVN of the hypothalamus of WT mice underwent three consecutive days of axial compression loading of tibiae or control shame load. Scale bar, 50 μm. (G) Representative images of immunofluorescence staining and quantitative analysis of the TH+ cells in the PVN of the hypothalamus of *EP4*^*wt*^ and *EP4*_*Avil*_^−/−^ mice underwent three consecutive days of axial compression loading of tibiae. Scale bar, 50 μm. N ⩾ 5 per group. *P < 0.05, **P<0.01, and N.S. indicates not significant. Statistical significance was determined by Student’s t-test.

The EP4 receptor is a key receptor for PGE2 in peripheral sensory nerves, and its signaling maintains the balance of osteogenesis and adipogenesis in adult mice by regulating sympathetic nerve activity(33). To determine whether mechanical loading-induced bone formation is mediated by PGE2-EP4 signaling in sensory nerves, we generated sensory nerve-specific EP4 knockout mice (*EP4*_*Avil*_^−/−^) by crossing *EP4 floxed (EP4*^*wt*^) mice with *Advilin*-Cre mice. In the *EP4*^*wt*^ mice, the bone mass increased significantly after mechanical loading compared to the control group, as shown by μCT analysis. However, this effect was diminished in the *EP4*_*Avil*_^−/−^ mice (Fig. 4A). Similarly, by immunostaining of trabecular bone sections we found that mechanical loading was associated with a significantly greater number of Ocn^+^ osteoblastic cells in *EP4*^*wt*^ mice compared to the unload control and these effects were absent in *EP4*_*Avil*_^−/−^ mice (Figures 4B).

Osteoblast-derived PGE2-mediated activation of EP4 receptors in peripheral sensory nerves promotes bone formation by inhibiting sympathetic activity via hypothalamic CREB signaling (32). By immunostaining of hypothalamic sections we found that mechanical loading was associated with significantly greater levels of phosphorylated CREB (pCREB) in the arcuate nucleus (ARC) compared with the unloaded control group (Figure 4C). In contrast, the hypothalamic pCREB levels in the ARC in the *Cox2*_*Ocn*_^−/−^ mice were not greater after mechanical loading compared with the *Cox2*^*wt*^ mice (Figure 4D). These data further demonstrate that osteoblasts secrete PGE2 in response to mechanical loading to activate skeletal interoception. Moreover, in the *EP4*_*Avil*_^−/−^ mice, the hypothalamic pCREB levels in the ARC was not higher after mechanical loading compared with *EP4*^*wt*^ control mice (Figure 4E), suggesting that mechanical loading may stimulate bone formation by activating a PGE2-EP4 ascending interoceptive pathway.

To evaluate sympathetic nerve activity, we performed immunostaining for tyrosine hydroxylase (TH), the rate-limiting enzyme in the synthesis of catecholamines, in the paraventricular nucleus (PVN) of the hypothalamus. TH expression was significantly lower in the mechanical loading group compared to the control group (Figure 4F). As expected, mechanical loading was associated with lower TH expression in the PVN of the *EP4*^*wt*^ mice but not the *EP4*_*Avil*_^−/−^mice (Figure 4G). Taken together, these findings suggest that skeletal interoception in response to mechanical loading is induced by CREB phosphorylation in the ARC, which inhibits TH expression in the PVN to promote osteogenesis.

### Mechanical loading signals through the ARC to regulate sympathetic activity

**Figure 5.**
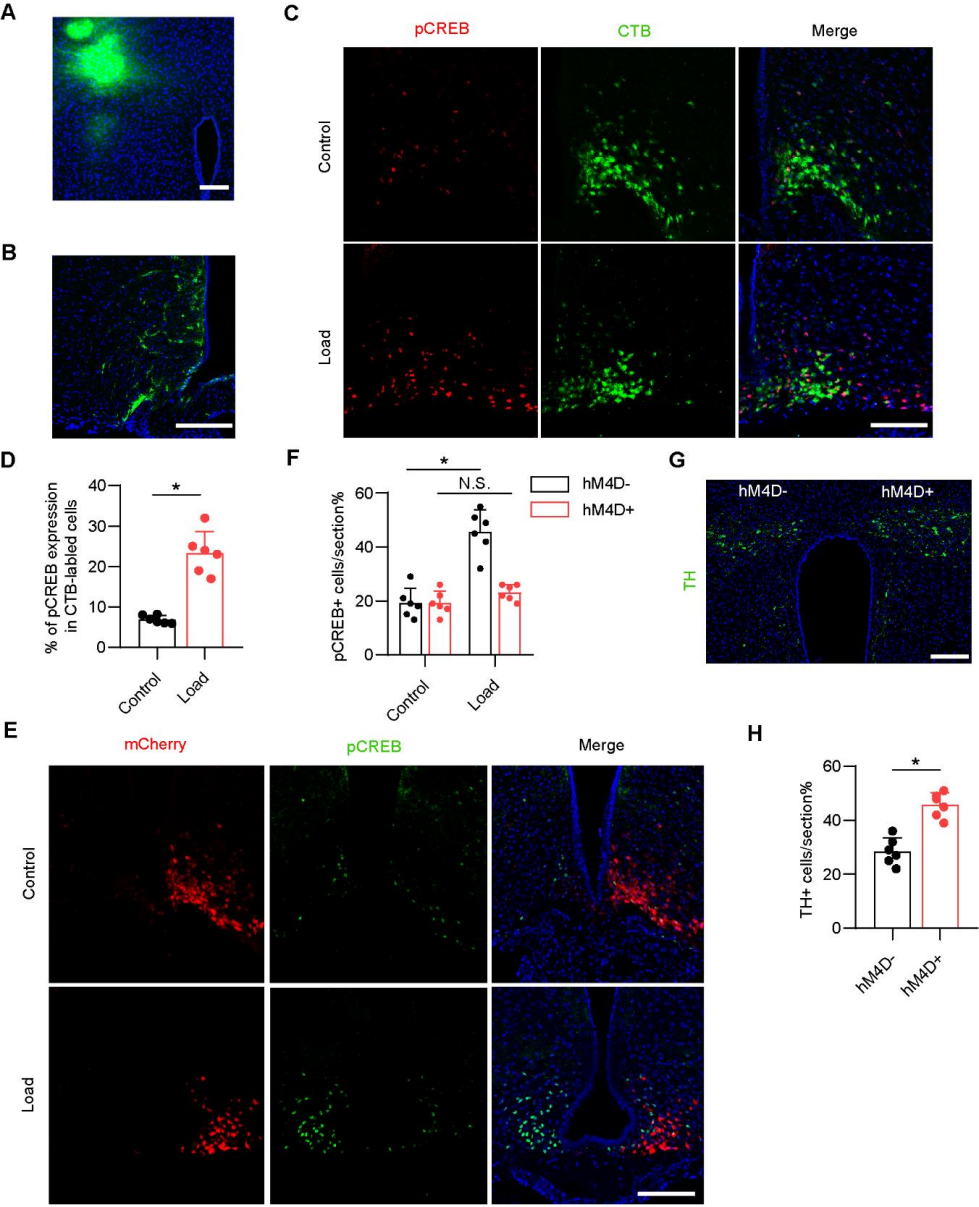
Mechanical load via hypothalamus AgRP neurons to regulate sympathetic activity. (A) Representative images of the CTB injection site in the PVN area of the WT mice. (B) Representative images of CTB+ neurons in the ARC of the hypothalamus after CTB injection in the PVN for 5 days. (**C**, **D**) Representative images of immunofluorescence staining (C) and quantitative analysis of the pCREB (red) and CTB (green) (D) in the ARC of the hypothalamus of WT mice underwent three consecutive days of axial compression loading of tibiae or control sham load after CTB injection in the PVN for 5 days. Scale bar, 50 μm. (**E**-**H**) AgRP-Ires-cre mice injected with pAAV-hSyn-DIO-hM4d(Gi)-mCherry in one side of the ARC (right) and control AAV in the other side (left). Mice injected saline or CNO (0.3 mg/kg of body weight, i.p.) before loading. (E, F) Representative images of immunofluorescence staining (E) and quantitative analysis (F) of the pCREB (green) and mCherry (red) in the ARC of hypothalamus of AgRP-Ires-cre mice underwent three consecutive days of axial compression loading of tibiae or control shame load. Scale bar, 50 μm. (G, H) Representative images of immunofluorescence staining (G) and quantitative analysis of the TH (green) (H) in the PVN of hypothalamus of AgRP-Ires-cre mice that underwent three consecutive days of axial compression loading of tibiae. Scale bar, 50 μm. N ⩾ 5 per group. *P < 0.05, **P<0.01, and N.S. indicates not significant. Statistical significance was determined by Student’s t-test.

The ARC is an essential site for the control of energy balance and the autonomic nervous system. The PVN has been identified as the primary hypothalamic site that contributes to the control of sympathetic outflow and energy expenditure(38). In the ARC, we have previously found that stimulation of skeletal interoception downregulates hypothalamic NPY expression to mediate lipolysis in the adipose tissue to liberate fatty acids to provide fuel for anabolic bone formation. To demonstrate that the ARC-PVN neural circuit controls energy balance, we injected the retrograde neural tracer cholera toxin b subunit (CTb) into the PVN of mice and found many CTb-labeled neurons in the ARC (Figure 5 A–B), suggesting ARC neurons project to the PVN. More importantly, mechanical loading was associated with significantly greater expression of pCREB in CTb-labeled neurons in the ARC compared to the unloaded control group (Figure 5 C–D). This combined data suggest an ARC-PVN circuit is involved in mechanical loading-induced skeletal interoception.

Agouti-related peptide (AgRP)^+^ neurons, which are primarily located in the ARC, express neuropeptide Y (NPY) and gamma-aminobutyric acid (GABA) to decrease energy expenditure via the sympathetic nervous system (39). To test whether AgRP^+^ neuron activity is sufficient to inhibit TH expression in the PVN, we chemogenetically inhibited AgRP^+^ neurons during mechanical loading by injecting a Cre-dependent adeno-associated virus (pAAV-hSyn-DIO-hM4d(Gi)-mCherry) unilaterally into the ARC. The infected AgRP^+^ neurons express inhibitory designer receptors hM4d, which can be inhibited following injection of the designer ligand clozapine-N-oxide (CNO). Inhibition of AgRP^+^ neurons on the right side of the brain was associated with significantly lower pCREB expression in the ARC during mechanical loading compared with the control left side of the brain (Figure 5E, F). Importantly, the suppression of TH expression in the PVN after mechanical loading was abolished in the AAV injection side (right side) in the ARC compared with the control (left) side (Figure 5G, H). Thus, these results suggest that mechanical loading activates an ARC-PVN circuit to tone down sympathetic nerve activity.

### Mechanical loading induces expression of the transcriptional repressor ICER via a PGE2-EP4 ascending interoceptive pathway

**Figure 6.**
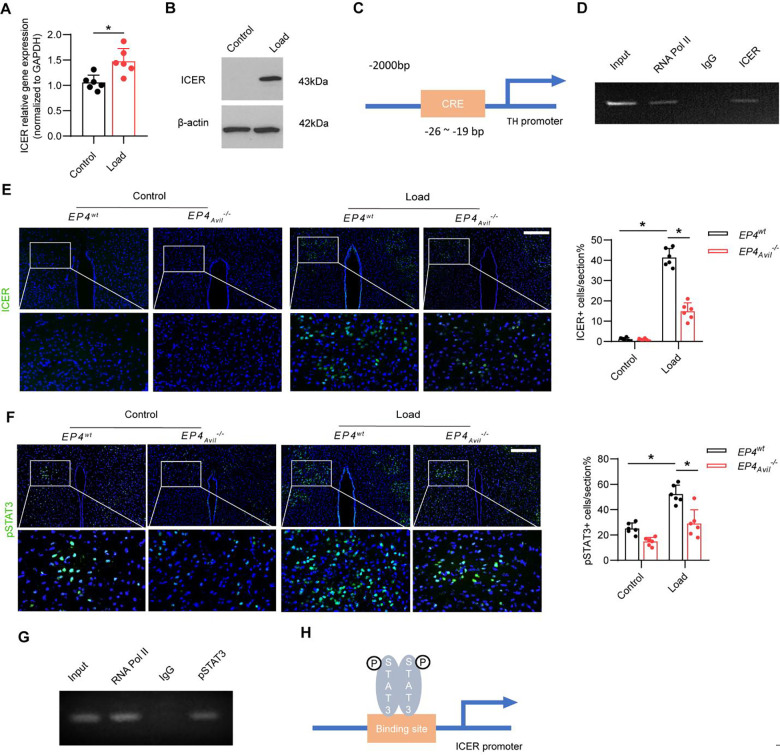
Mechanical load suppresses sympathetic activity through the ARC → PVN circuit via PGE2/EP4 interoceptive pathway. (A) RT-PCR quantitative analysis of *Crem* gene expression in the PVN area of the hypothalamus of WT mice underwent three consecutive days of axial compression loading of the tibia or control sham load. (B) Western blot analysis expression of ICER in the PVN area of the hypothalamus of WT mice underwent three consecutive days of axial compression loading of tibiae or control sham load. (C) Diagram of potential ICER binding site on the TH gene promoter. (D) ChIP analysis of ICER on TH gene promoter in the PVN area of WT mice underwent three consecutive days of axial compression loading of the tibia. (E) Representative images of immunofluorescence staining and quantitative analysis of the ICER+ cells in the PVN of the hypothalamus of *EP4*^*wt*^ and *EP4*_*Avil*_^−/−^ mice underwent three consecutive days of axial compression loading of tibiae or control sham load. Scale bar, 50 μm. (F) Representative images of immunofluorescence staining and quantitative analysis of the pSTAT3+ cells in the PVN of the hypothalamus of *EP4*^*wt*^ and *EP4*_*Avil*_^−/−^ mice underwent three consecutive days of axial compression loading of tibiae or control sham load. Scale bar, 50 μm. N ⩾ 5 per group. *P < 0.05, **P<0.01, and N.S. indicates not significant. Statistical significance was determined by Student’s t-test for A. Statistical significance was determined by two-way analysis of variance for E, F. (G) ChIP analysis of pSTAT3 on ICER gene promoter in the PVN area of WT mice underwent three consecutive days of axial compression loading of tibiae. (H) Diagram of the mechanism of mechanical load up-regulated ICER gene expression in the PVN area.

The inducible cAMP early repressor (ICER) is an endogenous repressor of cAMP-responsive element (CRE)-mediated gene transcription. To determine whether mechanical loading increases the expression of ICER in the hypothalamus to regulate *Th* gene expression, we measured *Crem* (the gene that encodes ICER) mRNA expression in the PVN in mice after mechanical loading through reverse transcription-polymerase chain reaction (RT-PCR). *Crem* mRNA expression was significantly higher in loaded mice compared to the unloaded control group (Figure 6A). By Western blot analysis, we also found greater ICER protein expression in the hypothalamus of loaded mice than in the unloaded control mice (Figure 6B). To investigate the transcriptional mechanism regulating this difference, we performed chromatin immunoprecipitation (ChIP) assay on the cAMP-responsive element (CRE) binding site in the *Th* gene promoter (Figure 6C) and found that mechanical loading induced specific binding of ICER to the CRE binding site of the *Th* promoter (Figures 6D). Moreover, to examine whether mechanical loading induced ICER expression through the PGE2-EP4-mediated skeletal interoception pathway, we analyzed the levels of ICER expression in the PVN of loaded and control in the *EP4*_*Avil*_^−/−^ and *EP4*^*wt*^ mice. By immunostaining of hypothalamic sections we found that expression of ICER was significantly greater in the PVN of loaded *EP4*^*wt*^ mice compared to the unloaded *EP4*^*wt*^ group. And the load-induced increase of ICER expression was drastically reduced in the *EP4*_*Avil*_^−/−^ mice (Figures 6E). Taken together, these results indicate that mechanical loading activates PGE2-EP4-mediated skeleton interoceptive signaling via an ARC-PVN circuit to induce ICER expression in the PVN to suppress *Th* expression and, thus, sympathetic drive.

Phosphorylated STAT3 (p-STAT3) acts as a transcription factor that binds to and regulate the expression of target genes, such as *POMC*, to regulate energy metabolism. We found that mechanical loading induced significant greater levels of p-STAT3 in the PVN in the *EP4*^*wt*^ mice (Fig. 6F). We next performed a ChIP assay of the potential pSTAT3 binding site in the *Crem* gene promoter and found that mechanical loading induced specific binding of pSTAT3 to the promoter (Figures 6G, H). In addition, to examine whether mechanical loading promotes pSTAT3 expression via the PGE2-EP4-mediated skeletal interoception pathway, we analyzed the levels of pSTAT3 expression in the PVN of the *EP4*_*Avil*_^−/−^ and *EP4*^*wt*^ mice. By immunostaining of hypothalamic sections we found that expression of pSTAT3 was significantly higher in the PVN of loaded *EP4*^*wt*^ mice compared to the unloaded *EP4*^*WT*^ mice and the stimulation was significantly attenuated in *EP4*_*Avil*_^−/−^ mice (Figures 6E) Therefore, our data show that mechanical loading activates a PGE2-EP4-mediated skeleton interoceptive pathway to induce activation of PVN pSTAT3 signaling to stimulate the synthesis of ICER.

### Aberrant alterations in PGE2 level leads to ankle osteoarthritis and pain

**Figure 7.**
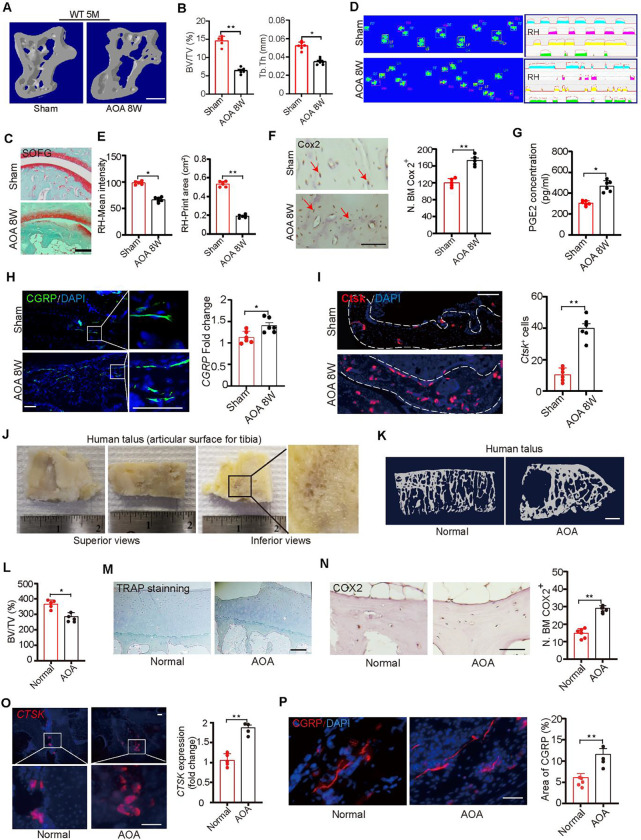
Alteration in PGE2 level leads to Ankle Osteoarthritis and pain through sensory nerve. (A, B) Representative μCT images (A) and quantitative analysis of trabecular bone fraction (BV/TV) and trabecular bone thickness (Tb. Th) of talus (B) from 20-week-old C57BL/6 mice with sham or ankle osteoarthritis surgery(AOA) for 8 weeks. Scale bars, 50 μm. (C) Representative images of Safranin Orange and fast green staining in the subchondral bone of talus from 20-week-old C57BL/6 mice with sham or ankle osteoarthritis surgery(AOA) for 8 weeks. Scale bars, 50 μm. (D, E) Representative images of catwalk (D) and ink blot analysis (E) of ipsilateral intensity and contact area of right hind paw of 20-week-old C57BL/6 mice with sham or ankle osteoarthritis surgery(AOA) for 8 weeks. (F) Representative images of immunostaining and quantitative analysis of the Cox2^+^ (brown) cells in the subchondral bone of talus of 20-week-old C57BL/6 mice with sham or ankle osteoarthritis surgery (AOA) for 8 weeks. Scale bars, 50 μm. (G) ELISA analysis of PGE2 level in the talus of 20-week-old C57BL/6 mice with sham or ankle osteoarthritis surgery (AOA) for 8 weeks. (H) Representative images of immunofluorescence staining and quantitative analysis of the CGRP+ sensory nerves (green) in the subchondral bone of talus of 20-week-old C57BL/6 mice with sham or ankle osteoarthritis surgery(AOA) for 8 weeks. Scale bars, 50 μm. (I) Representative images of immunostaining of Ctsk and quantitative analysis of Ctsk+ cells (red) in the subchondral bone of talus of 20-week-old C57BL/6 mice with sham or ankle osteoarthritis surgery(AOA) for 8 weeks. Scale bars, 50 μm. (J)Representative images of human talus samples from end stage of AOA patients with total ankle arthroplasty (TAA). (K, L) Representative μCT images (K) and quantitative analysis (L) of trabecular bone fraction (BV/TV) of healthy talus and end-stage AOA patient talus. Scale bars, 500 μm. (M) Representative images of immunostaining of TRAP in the subchondral bone of healthy talus and end-stage AOA patient talus. Scale bars, 50 μm. (N) Representative images of immunostaining and quantitative analysis of the COX2+ cells (brown) in the subchondral bone of healthy talus and end-stage AOA patient talus. Scale bars, 50 μm. (O) Representative immunofluorescence staining and quantitative analysis of CTSK (red) in the subchondral bone of healthy talus and end-stage AOA patient talus. Scale bars, 50 μm. (P) Representative images of immunofluorescence staining and quantitative analysis of the CGRP+ sensory nerves (red) in the subchondral bone of healthy talus and end-stage AOA patient talus. Scale bars, 50 μm. N ⩾ 5 per group. *P < 0.05, **P<0.01, and N.S. indicates not significant. Statistical significance was determined by Student’s t-test.

We next tested whether sensory innervation is initiated by PGE2 and is associated with ankle osteoarthritis (AOA)-associated pain. In mice, we excised the calcaneofibular ligament, the anterior talofibular ligament and the lateral ankle capsule (lateral model), an established mouse model of AOA and ankle pain. Both bone marrow (BV/TV) and trabecular bone parameters were significantly lower in 12-week AOA mice (Figure 7A, B). By safranin O and fast green (SOFG) staining of the talus we found that a green-stained bone matrix surrounded the cavities in the talus of AOA mice (Figure 7C), suggesting marked degeneration of the subtalar joint 8 weeks after surgery. Furthermore, ink blot analysis revealed a significant disparity between the percentage of right hind paw ipsilateral intensity and contact area (Figure 7D, E) of the 2 limbs at 2 months after AOA surgery in wild-type mice relative to sham-surgery controls. The purple waveform represents the mean grip intensity when the right hind paw is walking. Cox2 expression in osteocytes was greater in the AOA than in the sham-surgery controls (Figure 7F). Importantly, the density of CGRP^+^ neurofilaments was markedly higher in AOA mice than in the the sham-surgery group (Figure 7H), and PGE2 levels in the talus of the AOA mice was higher than in the sham controls (Figure 7G), suggesting that surgery leads to an increase in mechanical loading that in turn leads to greater PGE2 levels. In addition, Ctsk expression in osteocytes was significantly greater in the AOA mice compared with sham-surgery mice (Figure 7I), suggesting that Ctsk activity is associated with CGRP^+^ sensory innervation in the talus.

To further test our hypothesis in human disease, we collected 8 surgery specimens from patients with end-stage AOA who underwent total ankle arthroplasty (TAA). These specimens were systemically scanned and analyzed by μCT. The images demonstrated the specimens included the serial sagittal sections (Figure 7J) and showed that during the end-stage AOA period there was significantly less bone volume compared with the healthy talus (Figure 7K, L). There was no difference in TRAP+ osteoclast expression between the healthy controls and the AOA samples in the talus (Figure 7M). Importantly, COX2 expression in osteocytes were significantly greater in the AOA samples compared to the healthy talus (Figure 7N). Osteocyte expression of CTSK at steady state have been reported in healthy humans (40), and here by immunostaining for CTSK in the talus from the normal and AOA groups we observed significantly greater CTSK expression in the subchondral bone of talus 8-week after AOA samples compared to the normal healthy control (Fig. 7O). Furthermore, by immunostaining of talus sections we found that there were more calcitonin gene-related peptide (CGRP)^+^ sensory nerve fibers in the AOA human samples compared to the normal healthy talus (Figure 7P). Together, these results suggest that elevated PGE2 is associated with AOA-related pain via sensory nerve induction.

## Discussion

Weight-bearing bones undergo constant remodeling or modeling by transforming mechanical signaling into biochemical signals in bone cells(41). The mechanobiology of bone remodeling and structure has been well studied at the molecular and cellular level(15). However, it is unclear whether the mechanical signals of the bone cells are integrated at a physiological level and processed and regulated in the brain via interoception. Here, we found that the bone PGE2 levels are proportional to their weight bearing. When weight bearing changes, for example, via tail-suspension in mice, the PGE2 concentrations in various bones change according to changes in their weight bearing. Moreover, deletion of *Cox2*, and thus elimination of Pge2 expression, in osteoblast lineage cells or knockout of *Ep4* in sensory nerve blunts bone formation in response to mechanical loading. And sensory denervation also significantly reduces mechanical loading-induced bone formation. Moreover, mechanical loading induces CREB phosphorylation in the ARC to inhibit sympathetic *Th* expression in the PVN, thus promoting osteogenesis. Therefore, our data demonstrate that the hypothalamus perceives elevation of bone PGE2 in response to mechanical loading to maintain bone remodeling, structure and homeostasis.

Bone formation during remodeling under mechanical loading is an energy intensive process consuming glucose, ATP, oxygen and free fatty acids(42–44). Therefore, it is important to provide the resources from different organs for mechanical-induced bone remodeling. We found local mechanical loading induced a significant increase in pCREB expression in the ARC , which was blocked by conditional knock out of osteoblast *Cox2* or sensory *Ep4*. This suggests that mechanical loading acts along a PGE2-EP4 sensory axis to transmit the change in peripheral status to the hypothalamus to promote a compensatory response. In this manner, proper skeletal interoception is achieved.

We previously showed that elevated PGE2 concentrations in the bone marrow activated the expression of pCREB and the hypothalamic transcriptional co-repressor SMILE. SMILE and pCREB form a heterodimer to bind to the *Npy* promoter to downregulate the expression of hypothalamic NPY (45) and in turn induce adipose tissue lipolysis for osteoblastic bone formation (34). Thus, the activation of pCREB after mechanical loading suggests that this stimuli promotes the downregulation of hypothalamus NPY signaling to coordinate the fuel needed for proper bone synthesis. In this way, AgRP^+^ neuron-expressed NPY in the ARC plays a critical role in the central control of bone homeostasis.

TH^+^ neurons are highly prevalent in the PVN and are key regulators of sympathetic activity (46). We previously reported that downregulation of TH upon elevated PGE2 levels reduced sympathetic activity outflow to allow for the greater osteoblastic differentiation of mesenchymal stem/stromal cells (MSCs) and increased bone formation (33). Here, we found that mechanical loading suppresses TH expression in the PVN. Applying the retrograde neural tracer CTb, we found that ARC neurons project to the PVN and that specifical chemogenetic inhibition of AgRP^+^ neurons in the ARC significantly blocks mechanical loading-induced TH suppression in the PVN. These data suggest a mechanical loading-ARC (AgRP)-PVN (TH) circuitry acts upstream to regulate TH expression to mediate the mechanical loading interoceptive pathway.

We further identified the mechanistic pathway in the PVN that regulates *Th* expression by ChIP assay. Notably, we found that mechanical loading promotes the elevation of pSTAT3 levels in the PVN, perhaps via the ARC neural circuitry, and that the elevated pSTAT3 in turn binds to the promoter region of *Crem*, the gene that encodes the inducible cAMP early repressor, ICER, elevating its expression. ICER, in turn, binds to the promoter of *Th* to suppress its expression, thus suppressing sympathetic tone to promote bone formation. Together, these findings show that hypothalamic regulation of NPY and TH activity in response to mechanical loading coordinates energy metabolism with bone structure remodeling. Thus, the brain is essential for the control of mechanical loading-induced bone formation.

The talus in the ankle bears the highest body weight(47). Ankle osteoarthritis is known to be very painful(48). Compared to bones in other body locations, we found that under homeostatic conditions in adult mice the talus contains the highest PGE2 concentration, bone volume fraction and trabecular and cortical thickness. Tail suspension for 7 days dramatically reduced PGE2 levels, as well as bone mass and cortical bone, in the talus, indicating an active remodeling process in this bone in response to changes in mechanical loading. Interestingly, there are barely detectable TRAP^+^ osteoclasts in the talus from either healthy or AOA mice talus or in talus samples from humans with AOA. Bone remodeling is initiated by osteoclastic bone resorption, which is coupled with osteoblastic bone formation(49–51). This raises a question of how bone resorption is processed in talus remodeling without osteoclasts. By immunostaining, we found a much higher number of Ctsk^+^ osteocytes in mouse talus than in the calcaneus and tibia. The number of Ctsk^+^ osteocytes in talus were significantly increased after 7 days of TS or 8 weeks after AOA surgery, and this increase is accompanied by bone loss. Similarly in the human talus, we detected no TRAP^+^ osteoclasts from either normal human or from patients with AOA. However, CTSK was expressed in both normal and AOA talus, and its levels were significantly higher in the AOA talus and accompanied by significant bone loss compared to the healthy controls. This suggests that in the talus CTSK^+^ osteocytes likely perform the resorptive function usually mediated by osteoclasts. Indeed, the sympathetic nervous system promotes osteocyte-driven bone loss by secretion of extracellular vesicles containing bone-degrading enzymes for perilacunar bone resorption during lactation (52). It appears that skeletal interoception promotes osteocyte-mediated bone resorption primarily in weight-bearing bones, including cortical bone, whereas osteoclast-mediated bone remodeling occurs in the trabecular bone or in bones that bear less weight. AOA presents a condition that causes pain and disability, and currently, there is no disease-modifying treatment other than orthopedic surgery(53). The finding of high levels of PGE2 in the talus to maintain its homeostasis through skeletal interoception may explain the painful nature of AOA and shed light on a potential therapy. These findings, also show that, like tissue remodeling after injury and fibrosis, pathological conditions can occur when a homeostatic process occurs chronically.

In summary, our results show that the hypothalamus regulates bone remodeling and structure by perceiving elevated bone PGE2 concentrations that occur in response to mechanical loading. The axis of mechanical loading—PGE2-EP4 signaling—ARC—PVN—SNS acts as a skeletal interoceptive ascending pathway to transmit and process peripheral signals to the brain to promote bone remodeling. Our results suggest skeletal interoceptive regulation of osteocyte-mediated resorption for weight-bearing bones, including cortical bone, acts to maintain bone homeostasis upon mechanical loading. If this system acts chronically to excessive levels, such as in disease states, then this may explain the pain associated with such conditions, with implications for potential treatment.
